# Prenatal Depression Severity and Postpartum Care Utilization in a Medicaid Population

**DOI:** 10.1089/whr.2020.0079

**Published:** 2020-10-08

**Authors:** Susan G. Kornstein, Anny-Claude Joseph, Whitney C. Graves, Jordyn T. Wallenborn

**Affiliations:** ^1^ Department of Psychiatry, School of Medicine, Virginia Commonwealth University, Richmond, Virginia, USA.; ^2^ Institute for Women's Health, Virginia Commonwealth University, Richmond, Virginia, USA.; ^3^ Department of Biostatistics, School of Medicine, Virginia Commonwealth University, Richmond, Virginia, USA.; ^4^ Division of Epidemiology, Department of Family Medicine and Population Health, School of Medicine, Virginia Commonwealth University, Richmond, Virginia, USA.

**Keywords:** depression, mental health, Medicaid, postpartum, postpartum visit

## Abstract

***Background:*** Postpartum visits are a necessary continuum of medical care for women who are diagnosed with depression during pregnancy. However, postpartum care utilization is typically lower in populations who face adverse events and it is unclear to what extent having depression during pregnancy may compromise postpartum visit follow-up. Our study examined the association between severity of prenatal depression and postpartum care utilization among women on Medicaid.

***Materials and Methods:*** Data from a university-based, nonprofit managed care organization (2008–2012) were analyzed (*N* = 846). Prenatal depression severity and postpartum care utilization were determined using the International Classification of Diseases, Ninth Revision (ICD-9) codes, from medical claims records. Bivariate and multivariable logistic regression was conducted. Odds ratios and 95% confidence intervals (CIs) were calculated.

***Results:*** The majority (64.2%) of women received a mild/moderate prenatal depression diagnosis and 52.5% of the total sample attended their postpartum care visit. After adjusting for confounders, we found decreased odds of postpartum care utilization among women with less severe diagnoses. Women with a mild/moderate prenatal depression diagnosis were 12% less likely to attend the postpartum care visit compared with women with a severe prenatal depression diagnosis (adjusted odds ratio = 0.88, 95% CI = 0.65–1.19). However, this finding was not statistically significant.

***Conclusions:*** Our study did not yield evidence of a statistically significant relationship between prenatal depression severity and postpartum visit attendance among a sample of Medicaid beneficiaries. Additional research is needed to assess the association between prenatal depression severity and postpartum care use to enhance continuity of services for Medicaid-insured women into the postpartum period.

## Introduction

Prenatal depression is one of the most common mental disorders during pregnancy, impacting more than 10% of pregnant women in the United States.^1^ Prenatal depression can lead to poor outcomes for both mother and child, including low birth weight, poor infant psychosocial and cognitive functioning, premature breastfeeding cessation, and reduced mother–child connectedness.^2–4^ The heterogeneity of depression severity may also lead to differential health outcomes and health-seeking behaviors. Severe depression has been linked with decreased maternal–fetal attachment during pregnancy and has been indicated as impairing a woman's parental role.^5,6^ In addition, a history of depression has been shown to increase a woman's risk of postpartum depression by 20-fold.^7^

As a result of these varied negative effects, the promotion of depression screening during pregnancy has been highly considered in recent years.^2,3,8^ In 2015, the American College of Obstetricians and Gynecologists (ACOG) provided a recommendation to support prenatal depression screening at least once during the perinatal period.^8^ This recommendation was further supported by a 2016 report from the U.S. Preventive Services Task Force, which showed that screening could reduce depression among both pregnant and postpartum women.^9^

When incorporated into a prenatal care visit, depression screening may increase the likelihood that a patient obtains an early diagnosis and receives referrals to appropriate mental health providers and educational resources. In addition, this may also improve a practitioner's ability to provide continued treatment from pregnancy through the postpartum period. Yet, there is scant literature that has explored outcomes associated with depression diagnoses during pregnancy and continuity of care postdelivery.^10^ Furthermore, little is known about the severity of depression and utilization of health care services.

Research has shown severe disparities in postpartum care utilization. The likelihood of attending a postpartum care visit differs significantly by demographic factors (*i.e.*, age, race/ethnicity, marital status), socioeconomic status (*i.e.*, income, insurance), pregnancy intendedness, prenatal care utilization, and a history of poor experiences with medical care.^11–14^ Research has also shown that Medicaid recipients are less likely to attend a postpartum care visit than recipients of private or other types of insurance. DiBari et al. reported that women who were Medicaid recipients were twice as likely (odds ratio [OR] = 2.2; 95% confidence interval [CI] = 1.5–3.1) to not attend their postpartum visit compared with women with private insurance.^11^ A study that used Managed Care Organization data also reported that nearly 51% of Medicaid-insured women did not attend their postpartum visit.^15^

Lack or inadequate postpartum visit attendance or utilization seriously compromises continuity of care for women who are diagnosed with depression during pregnancy. In states that have not yet expanded Medicaid, some women may lose their insurance shortly after birth and may not have other means to access services.^16^ This may have a serious detrimental effect on the care of postpartum women. In response, additional research is needed to examine the utilization of adequate and timely postpartum health services in women at risk of postpartum depression. With the adverse effects posed by a perinatal depression diagnosis and the need for continued treatment and follow-up during pregnancy, it is important to understand the service-seeking behavior of Medicaid-insured women with a prenatal depression diagnosis. Therefore, the aim of our study was to examine the association between severity of a prenatal depression diagnosis and postpartum care utilization among women enrolled in Medicaid through a managed care organization in Virginia.

## Materials and Methods

The current study used data from Virginia Premier Inc., a university-based, nonprofit managed care organization. Data include women with singleton births between 2008 and 2012 collected from medical claims and interviews by case managers (*N* = 25,692). Additional information on Virginia Premier, Inc. can be found elsewhere. The current study restricted the analysis to *N* = 846 women who received a depression diagnosis during a prenatal care visit, who had information on the severity of their depression, and who were not indicated as being in partial or full remission.

The main outcome, postpartum care utilization (yes; no), was identified using the International Classification of Diseases, Ninth Revision, Clinical Modification (ICD-9-CM) codes extracted from the medical claims data. ICD-9 code V24.2 was used to assess the occurrence of postpartum care utilization. The exposure of interest, severity of a single or recurrent prenatal depression diagnosis at a prenatal care visit, was categorized as mild/moderate (ICD-9 codes 296.21, 296.31, 296.22, or 296.32) or severe (ICD-9 codes 296.23, 296.24, 296.33, or 296.34). Mild and moderate diagnoses were grouped together due to the small sample size of our study population and the low prevalence of women with mild diagnoses within our study sample (less than 10%). In addition, women who had claims for both single and recurrent episodes of prenatal depression were classified as having a diagnosis of severe depression for this analysis.

Several demographic and health behavior factors were considered potential confounders. Sociodemographic characteristics included maternal race/ethnicity (white, black, Hispanic, and other), age (<18; 18–24; 25–34; ≥34 years), and maternal educational attainment (less than high school, high school graduate, more than high school). The predominant type of health care system utilized, termed location for majority of medical services, was categorized as “private office,” “hospital,” or “health department or federally qualified health centers (FQHC).” Health behavior factors included tobacco use before or during pregnancy (yes; no), history of alcohol abuse/dependence (yes; no), and substance abuse/dependence before or during pregnancy (yes; no). Other factors assessed included birth control use (yes; no), pregnancy complications (yes; no), and previous preterm birth (yes; no). Pregnancy complications included hypertension, diabetes, anemia, cervical incompetence, ectopic pregnancy, uterine inertia, premature separation of placenta, and placenta previa.

Categorical measures were summarized with frequencies and percentages and were assessed for differences in postpartum visit attendance status using chi-square tests. Continuous measures were summarized with means and standard deviations and were assessed for differences in the study outcome using *t*-tests. Bivariate and multivariable logistic regression modeling was conducted to assess the relationship between prenatal depression severity and postpartum visit attendance. Covariates that resulted in a 10% or greater change in the crude odds ratio (COR) were considered for inclusion in the parsimonious adjusted regression model as confounders; however, no confounder met this inclusion criterion. Therefore, the parsimonious model included covariates identified as having statistically significant chi-square tests with both the targeted exposure and outcome of interest. A fully adjusted model with all identified potential confounders was also utilized. The fully adjusted model accounted for all covariates except race/ethnicity and maternal educational attainment due to their high percentage of missing in the study sample (>10%). The Akaike's Information Criterion (AIC) was used to compare model fit. All analyses were conducted using SAS software v 9.4 with a specified significance level of 0.05. This study was deemed exempt by the Institutional Review Board of Virginia Commonwealth University.

## Results

Over half of women (52.5%) attended their postpartum care visit. Approximately 64.2% of women had a mild/moderate depression diagnosis and 35.8% had a severe diagnosis. The majority of women were white (68.1%), between 18 and 34 years of age (88.7%), and had at least a high school education (72.1%). Ninety-four percent of participants used the hospital as their major health care system. With respect to health behaviors, more than half of women reported tobacco use (53.3%), 18.8% reported drug abuse/dependence, and 6.2% reported alcohol abuse/dependence. Twenty percent of women were currently using birth control. Nearly a third of women reported pregnancy complications (32.5%) and one percent of women had a prior preterm birth. A statistically significant difference was found between age, tobacco use, birth control use, and postpartum care visit attendance (Table 1).

**Table 1.** Distribution of Population Characteristics by Postpartum Care Visit Attendance

	Total population (N = 846)	Postpartum care visit		
		Yes (n = 444) (52.5%)	No (n = 402) (47.5%)	p^a^
Severity of depression diagnosis				0.4747
Mild/moderate	543 (64.2)	280 (63.1)	263 (65.4)	
Severe	303 (35.8)	164 (36.9)	139 (34.6)	
Age				0.0047^b^
12–17 Years	31 (3.6)	22 (5.0)	9 (2.2)	
18–24 Years	368 (43.5)	209 (47.1)	159 (39.6)	
25–34 Years	382 (45.2)	187 (42.1)	195 (48.5)	
≥35 Years	65 (7.7)	26 (5.9)	39 (9.7)	
Mean (SD)	25.8 (5.5)	25.1 (5.4)	26.6 (5.6)	<0.0001^b,c^
Race^d^				0.0716
White	320 (68.1)	163 (63.7)	157 (73.4)	
Black	101 (21.5)	64 (25.0)	37 (17.3)	
Other	49 (10.4)	29 (11.3)	20 (9.4)	
Education^d^				0.6178
Less than high school	80 (28.0)	48 (27.4)	32 (28.8)	
High school	146 (51.1)	87 (49.7)	59 (53.2)	
More than high school	60 (21.0)	40 (22.9)	20 (18.0)	
Location for majority of services				0.1096
Private	12 (1.4)	6 (1.4)	6 (1.5)	
Hospital	802 (94.8)	427 (96.2)	375 (93.3)	
Health department/FQHC	32 (3.8)	11 (2.5)	21 (5.2)	
Tobacco use				0.0178
No	396 (46.8)	225 (50.7)	171 (42.5)	
Yes	450 (53.2)	219 (49.3)	231 (57.5)	
Drug abuse/dependence				0.6663
No	687 (81.2)	363 (81.8)	324 (80.6)	
Yes	159 (18.8)	81 (18.2)	78 (19.4)	
Alcohol abuse/dependence				0.1771
No	794 (93.9)	412 (92.8)	382 (95.0)	
Yes	52 (6.2)	32 (7.2)	20 (5.0)	
Birth control use				<0.0001^b^
No	673 (79.6)	330 (74.3)	343 (85.3)	
Yes	173 (20.5)	114 (25.7)	59 (14.7)	
Pregnancy complications				0.2594
No	571 (67.5)	292 (65.8)	279 (69.4)	
Yes	275 (32.5)	152 (34.2)	123 (30.6)	
Previous preterm birth				0.2812
No	835 (98.7)	440 (99.1)	395 (98.3)	
Yes	11 (1.3)	4 (0.9)	7 (1.7)	

^a^ Chi-square test.

^b^ Indicates significance at *α* = 0.05.

^c^ *T*-test.

^d^ Percent missing greater than 10%.

FQHC, federally qualified health centers; SD, standard deviation.

Logistic regression models did not indicate a statistically significant relationship between severity of depression diagnosed at the prenatal care visit and postpartum care utilization (Table 2). The unadjusted analysis revealed that participants diagnosed with mild/moderate depression were 10% less likely to attend their postpartum visit compared with those who were diagnosed with severe depression at their prenatal care visit (COR = 0.90, 95% CI = 0.68–1.20). In the adjusted analysis, the fully adjusted model displayed better fit (AIC = 1150.61) than the parsimonious model that only controlled for maternal age and tobacco use (AIC = 1164.66). In the fully adjusted model, the odds of postpartum care utilization were 12% lower among women who were diagnosed with mild/moderate depression compared with women diagnosed with severe depression at the prenatal care visit (adjusted odds ratio = 0.88, 95% CI = 0.66–1.19).

**Table 2.** Association Between Severity of Prenatal Depression Diagnosis and Postpartum Visit Attendance

Prenatal depression diagnosis	COR^a^ (95% CI)	AOR^b^ (95% CI)	AOR^c^ (95% CI)
Mild/moderate	0.90 (0.68–1.20)	0.86 (0.65–1.15)	0.88 (0.66–1.19)
Severe	1.00	1.00	1.00

^a^ Crude model; AIC = 1174.21.

^b^ Parsimonious model adjusted for maternal age and tobacco use; AIC = 1164.66.

^c^ Full model adjusted for maternal age, location for majority of services, tobacco use, drug use/dependence, alcohol use/dependence, birth control use, pregnancy complications, and previous preterm birth; AIC = 1150.61.

AIC, Akaike's Information Criterion; AOR, adjusted odds ratio; CI, confidence interval; COR, crude odds ratio.

## Discussion

The current study did not yield evidence of a statistically significant relationship between prenatal depression severity and postpartum visit attendance, although a potential decreased use of postpartum care was exhibited among women with less severe depression diagnoses. Moreover, postpartum care utilization among our study sample was significantly lower than the national average at a prevalence of ∼53%. According to the U.S. Department of Health and Human Services (2013), the national prevalence of postpartum visit attendance among women in the United States, regardless of insurance status, was 90%.^17^

The majority of previous quantitative studies that examined postpartum care use have not assessed prenatal mental health factors as predictors for postpartum care utilization.^11–14,18^ To our knowledge, only one previous study investigated depression as a correlate and postpartum visit attendance among Medicaid beneficiaries. A cross-sectional study conducted in Virginia reported that women with a depression history had increased odds of postpartum visit attendance, although this finding was not statistically significant.^15^ Nonetheless, mental health-related themes have emerged in several qualitative studies examining postpartum care utilization. Bennett et al.^19^ conducted semistructured interviews among 22 women with a gestational diabetes mellitus diagnosis to examine their experiences and perceived barriers/facilitators to postpartum visit attendance. This study identified that experiencing anxiety or being overwhelmed in the postpartum period was a barrier to seeking postpartum care. Conversely, Henderson et al.^12^ interviewed women representing a predominantly urban, Medicaid population (*N* = 20) to assess their postpartum care visit experiences and found that their visits were perceived as an opportunity to evaluate and maintain both their physical and mental health.^12^

The conflicting results from these studies may be due to the demographics of each sampled population, including mean age, and reported race/ethnicity. All participants in Bennett et al.^19^ had insurance coverage, but the type of coverage was not specified. On the contrary, Masho et al.^15^ and Henderson et al.^12^ engaged predominantly or complete Medicaid populations that are similar to the sample population of our study. Thus, the comparable positive relationship exhibited between mental health and postpartum care utilization may be specific to the targeted population of women enrolled in Medicaid.

Our finding that the odds of attending a postpartum visit were decreased among women with mild/moderate prenatal depression diagnoses compared with women with severe diagnoses may be explained by the relationship between health needs and services utilization. Andersen's Behavioral Model of Health Services Use (1995) is a commonly used conceptual model that incorporates health needs as one of its key constructs.^20^ Health needs, whether perceived or clinically diagnosed, are considered to play an important role in service utilization, as a person's individual perception of his/her own health status, symptoms, and related experiences may contribute to his/her decision to seek appropriate care. In addition, an individual's health needs may also facilitate help-seeking behaviors and subsequent health services use.^21^ This may explain why women with severe depression, and who were at an increased risk of experiencing adverse sequelae, may have been more likely to attend the postpartum visit.

Prenatal depression education might also explain our findings. Prior studies have shown that more than two-thirds of women have discussed what to do when experiencing signs of depression with a prenatal care provider.^22,23^ Receiving this information may have led women with severe diagnoses to be more intentional about receiving postpartum care based upon their symptoms and experiences compared with women with mild/moderate diagnoses. However, having a depression history is a key risk factor for postpartum depression.^7^ Therefore, it is important to ensure that all women experiencing prenatal depression, even those with less severe cases, understand the importance and benefits of maintaining continuity of maternal health care into the postpartum period. It would be beneficial for future studies to investigate potential mediators of this association (*e.g.*, postpartum depression, medication, or psychotherapy treatment after initial depression diagnosis) to further explain what is driving postpartum care utilization among this population.

To the authors' knowledge, our study is the first to examine the relationship between severity of a prenatal depression diagnosis and postpartum care utilization among a sample of low-income, high-risk women. Obtaining perspectives from Medicaid beneficiaries is essential to eliminating present maternal and child health disparities, as women receiving care through Medicaid are less likely to attend the postpartum care visit and may be at a higher risk for a host of health conditions.^11,19^ The use of medical claims data also reduces the potential for recall bias because the information was not self-reported. However, there are several limitations. Because the sample population was limited to women covered by a managed care organization in the state of Virginia, these findings may not be generalizable to other populations. Potential confounding factors that could have influenced our measure of effect, such as maternal educational attainment, race/ethnicity, adequacy of mental health counseling/education, and social support, were not available in the data set or could not be assessed due to a high percentage of missing data. As a result, the analysis may be prone to omitted variable bias.

## Conclusions

The results of this study support prior evidence demonstrating the relationship between health needs and services utilization.^21,24^ Additional research is needed to assess the association between prenatal depression severity and postpartum care utilization among disparate groups of women, representing various demographic backgrounds and social classes. Due to the limited 2-month time period that some Medicaid beneficiaries have to receive postpartum care,^16,25^ more evidence is needed to support policy efforts that will improve access to adequate health coverage and encourage use of mental health services among low-income women. Subsequent studies should also examine the effects of mild and moderate depression separately on postpartum care utilization and the effectiveness of prenatal mental health screening and treatment adherence in facilitating the utilization of health services among women into the postpartum period. Additional understanding of these associations may provide novel insights to help increase postpartum care use among women who are less likely to attend their visit and improve access to mental health resources and treatment for high-risk women who may not otherwise seek care.

## Abbreviations Used

AIC: Akaike's Information Criterion
AOR: adjusted odds ratio
CI: confidence interval
COR: crude odds ratio
ICD-9: International Classification of Diseases, Ninth Revision
OR: odds ratio
SD: standard deviation

## References

[1] Mukherjee S, Trepka MJ, Pierre-Victor D, et al. Racial/ethnic disparities in antenatal depression in the United States: A systematic review. Matern Child Health J 2016;20:1780–1797 27016352 10.1007/s10995-016-1989-x

[2] Grote NK, Katon WJ, Russo JE, et al. Collaborative care for perinatal depression in socioeconomically disadvantaged women: A randomized trial. Depress Anxiety 2015;32:821–834 26345179 10.1002/da.22405 PMC4630126

[3] Molina KM, Kiely M Understanding depressive symptoms among high-risk, pregnant, African-American women. Womens Health Issues 2011;21:293–303 21565525 10.1016/j.whi.2011.01.008 PMC3488109

[4] Wallenborn JT, Joseph A-C, Graves WC, et al. Prepregnancy depression and breastfeeding duration: A look at maternal age. J Pregn 2018;2018:4825727 10.1155/2018/4825727 PMC6236915 30515328

[5] McFarland J, Salisbury AL, Battle CL, et al. Major depressive disorder during pregnancy and emotional attachment to the fetus. Arch Womens Ment Health 2011;14:425 21938509 10.1007/s00737-011-0237-z PMC3248759

[6] Foster CE, Webster MC, Weissman MM, et al. Course and severity of maternal depression: Associations with family functioning and child adjustment. J Youth Adolesc 2008;37:906–916 25013241 10.1007/s10964-007-9216-0 PMC4086840

[7] Silverman ME, Reichenberg A, Savitz DA, et al. The risk factors for postpartum depression: A population-based study. Depress Anxiety 2017;34:178–187 28098957 10.1002/da.22597 PMC5462547

[8] Byatt N, Levin LL, Ziedonis D, et al: Enhancing participation in depression care in outpatient perinatal care settings: A systematic review. Obstet Gynecol 2015;126:1048 26444130 10.1097/AOG.0000000000001067 PMC4618720

[9] O'Connor E, Rossom RC, Henninger M, et al: Primary care screening for and treatment of depression in pregnant and postpartum women: Evidence report and systematic review for the US Preventive Services Task Force. JAMA 2016;315:388–406 10.1001/jama.2015.18948 26813212

[10] Committee on Obstetric Practice. The American College of Obstetricians and Gynecologists Committee Opinion no. 630. Screening for perinatal depression. Obstet Gynecol 2015;125:1268–1271 25932866 10.1097/01.AOG.0000465192.34779.dc

[11] DiBari JN, Yu SM, Chao SM, et al. Use of postpartum care: Predictors and barriers. J Pregnancy 2014;2014:530769 24693433 10.1155/2014/530769 PMC3945081

[12] Henderson V, Stumbras K, Caskey R, et al. Understanding factors associated with postpartum visit attendance and contraception choices: Listening to low-income postpartum women and health care providers. Matern Child Health J 2016;20:132–143 27342600 10.1007/s10995-016-2044-7 PMC5290059

[13] Weir S, Posner HE, Zhang J, et al. Predictors of prenatal and postpartum care adequacy in a Medicaid managed care population. Womens Health Issues 2011;21:277–285 21565526 10.1016/j.whi.2011.03.001

[14] Wilcox A, Levi EE, Garrett JMJM, et al. Predictors of non-attendance to the postpartum follow-up visit. Matern Child Health J 2016;20:22–27 27562797 10.1007/s10995-016-2184-9

[15] Masho SW, Cha S, Karjane N, et al. Correlates of postpartum visits among Medicaid recipients: An analysis using claims data from a managed care organization. J Womens Health (Larchmt) 2018;27:836–843 29451839 10.1089/jwh.2016.6137

[16] American College of Obstetricians and Gynecologists: Weight gain during pregnancy. Committee opinion no. 548. Obstet Gynecol 2013;121:210–212 23262962 10.1097/01.aog.0000425668.87506.4c

[17] U.S. Department of Health and Human Services, Health Resources and Services Administration, Maternal and Child Health Bureau. Child Health USA 2013. Rockville, Maryland: U.S. Department of Health and Human Services, 2013

[18] Lu MC, Prentice J The postpartum visit: Risk factors for nonuse and association with breast-feeding. Am J Obstet Gynecol 2002;187:1329–1336 12439527 10.1067/mob.2002.126848 PMC4353609

[19] Bennett IM, Marcus SC, Palmer SC, et al. Pregnancy-related discontinuation of antidepressants and depression care visits among Medicaid recipients. Psychiatr Serv 2010;61:386–391 20360278 10.1176/ps.2010.61.4.386

[20] Andersen RM National health surveys and the behavioral model of health services use. Med Care 2008;46:647–653 18580382 10.1097/MLR.0b013e31817a835d

[21] Andersen RM Revisiting the behavioral model and access to medical care: Does it matter? J Health Soc Behav 1995;36:1–10 7738325

[22] Farr SL, Denk CE, Dahms EW, et al. Evaluating universal education and screening for postpartum depression using population-based data. J Womens Health (Larchmt) 2014;23:657–663 25072299 10.1089/jwh.2013.4586 PMC6732792

[23] Farr SL, Ko JY, Burley K, et al. Provider communication on perinatal depression: A population-based study. Arch Womens Ment Health 2016;19:35–40 25578631 10.1007/s00737-014-0493-9 PMC6085752

[24] Chang JJ, Tabet M, Elder K, et al. Racial/ethnic differences in the correlates of mental health services use among pregnant women with depressive symptoms. Matern Child Health J 2016;20:1911–1922 27126445 10.1007/s10995-016-2005-1

[25] Sonfield A, Pollack HAJJohp, policy, law: The Affordable Care Act and reproductive health: Potential gains and serious challenges. J Health Polit Policy Law 2013;38:373–391 10.1215/03616878-1966342 23262768

